# Inadvertent, Self-Induced Pneumoparotitis Provoking Pneumomediastinum

**DOI:** 10.7759/cureus.34016

**Published:** 2023-01-20

**Authors:** Emily R Youner, Robert Cox, Philip Zapanta

**Affiliations:** 1 Division of Otolaryngology - Head & Neck Surgery, The George Washington University, Washington, D.C., USA; 2 ENT and Allergy, Sovah Physician Practices, Danville, USA

**Keywords:** pneumoparotid, stensen's duct, parotid gland, pneumomediastinum, pneumoparotitis

## Abstract

Pneumoparotid refers to the presence of air within the parotid gland and pneumoparotitis indicates overlying inflammation or infection. Several physiologic mechanisms exist to prevent the reflux of air and oral contents into the parotid gland, however, these safeguards can be overcome by high intraoral pressures, thus provoking pneumoparotid. Whereas the relationship between pneumomediastinum and air dissecting up into cervical tissues is well understood, the relationship between pneumoparotitis and free air traveling downwards through contiguous structures within the mediastinum is less defined. We present a case of a gentleman who experienced the sudden onset of facial swelling and crepitus in the context of inflating an air mattress with his mouth, who was ultimately found to have pneumoparotid with consequent pneumomediastinum. Discussion of this unusual presentation is important to facilitate recognition and treatment of this uncommon pathology.

## Introduction

Pneumoparotid describes the presence of air within the parenchyma of the parotid gland; pneumoparotitis indicates inflammation or infection [1]. Air and oral flora are introduced into parotid acini by retrograde flow through Stensen’s duct, often due to puffing out one’s cheeks [2]. High intraoral pressure can provoke rupture of parotid acini and air extravasation within facial and cervical structures dissecting down into the mediastinum [2]. This extravasated air within the mediastinum can dissect up into surrounding tissue, including cervical subcutaneous spaces, causing crepitus on exam [3].

Under physiologic conditions, contraction of the buccinator muscle and the small opening relative to duct size prevent oral contents from refluxing into Stensen’s duct [1]. Typically, intraoral pressures measure 2-3 mmHg but can reach 140 mmHg during activities like glassblowing, creating conditions favorable to introduction of air and oral flora into the parotid gland [1]. Other possible etiologies of pneumoparotid that have been identified include unconscious habits, diseases that induce coughing, vomiting, or sneezing, continuous positive pressure, and iatrogenic causes such as dental and surgical procedures [4].

The relationship between pneumomediastinum and air dissecting into cervical tissues is well understood; pneumoparotitis leading to free air traveling downwards through contiguous structures within the mediastinum is less defined. Recognizing the unusual presentation is paramount to appropriate diagnosis and management. This study is compliant with ethical research standards and deemed exempt by The George Washington University Office of Human Research.

## Case presentation

We report a case of a 37-year-old male without history of parotid infection or surgery who presented to the Emergency Department with left-sided facial swelling after attempting to inflate his air mattress using his mouth. He denied any occupational or otherwise regular exposure to increased intraoral pressure, such as glassblowing. He reported sudden left facial crepitus while inflating the mattress, which progressed with increasing swelling over the next several hours. Also present were facial numbness, tingling, mild throat pain and odynophagia, along with pressure in his face, neck, and left ear. Physical exam revealed left facial asymmetry with swelling and crepitus overlying the left parotid gland and left lateral neck. Crepitus was palpable into the upper chest. Petechiae and ecchymosis were noted intraorally overlying Stensen’s duct. Computed tomography (CT) maxillofacial and thorax demonstrated significant subcutaneous emphysema infiltrating the parotid gland and the masticator, buccal, and submandibular spaces extending inferiorly through subcutaneous tissues of the neck with pneumomediastinum.

Evaluation by otolaryngology and thoracic surgery confirmed pneumoparotitis secondary to barotrauma with resultant pneumomediastinum. Given continued vital signs stability and relatively small amount of mediastinal air, the patient was discharged with prophylactic amoxicillin-clavulanate. He was seen in clinic the following week with marked improvement in edema and crepitus and without signs of infectious complication.

Investigations

**Figure 1:**
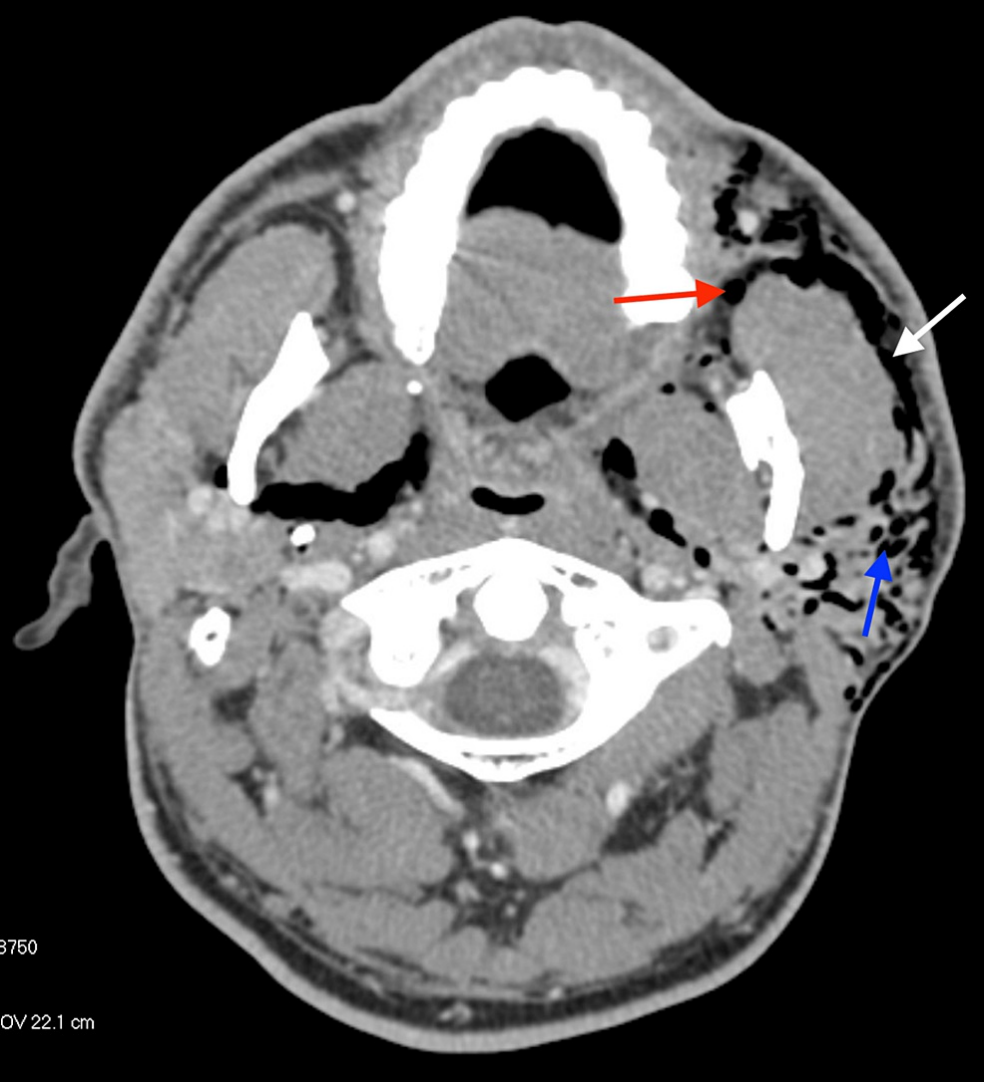
CT Maxillofacial with contrast, axial view CT Maxillofacial with contrast demonstrating air within the parotid gland extending subcutaneously through the soft tissues of the face and along the course of the parotid duct. Red arrow: air around Stensen’s duct. Blue arrow: air within parotid. White arrow: subcutaneous emphysema within soft tissues of face

**Figure 2:**
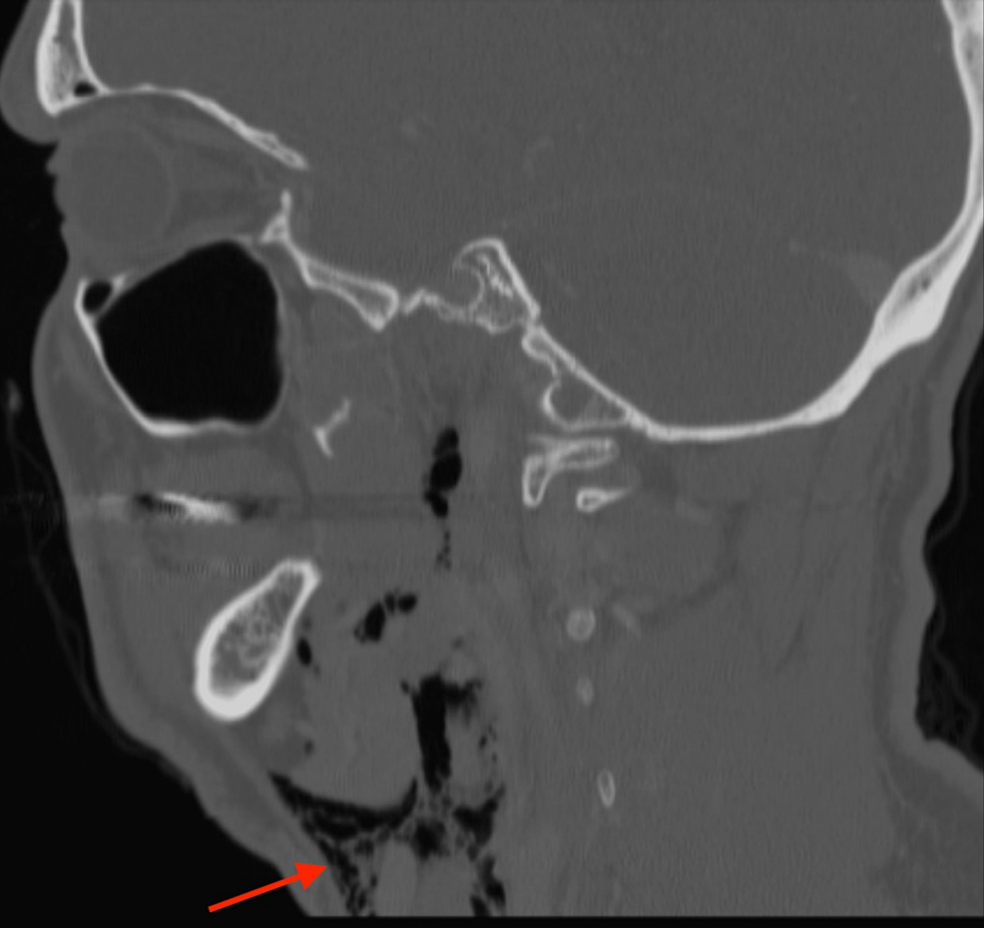
CT Maxillofacial, sagittal view Red arrow: subcutaneous emphysema extending inferiorly through the soft tissues of the neck

Investigation included maxillofacial CT scan with contrast, shown in Figure 1 and Figure 2. Figure 1 demonstrates air within the parotid gland extending subcutaneously through soft tissues of the face and along the parotid duct. Figure 2 reveals subcutaneous emphysema extending inferiorly through cervical soft tissues. 

## Discussion

Pneumoparotid presents with swelling, erythema, tenderness, and crepitus overlying the affected gland [5]. Due to acini rupture, air may extend beyond the parotid causing subcutaneous emphysema in the face, neck, and mediastinum [5]. Frothy saliva and air bubbles may be observed at the orifice of Stensen’s duct upon gland massage [5]. Purulence may indicate superimposed bacterial infection from refluxed oral flora.

Spontaneous pneumomediastinum is most often seen secondary to bronchial hyper-reactivity or barotrauma; pneumomediastinum seen as a complication of pneumoparotitis is rarely reported [5]. However, in the setting of pneumoparotitis without esophageal or tracheal rupture or pneumothorax, pneumoparotitis becomes the most likely etiology. In this case, CT findings further support pneumoparotitis as the etiology for pneumomediastinum, including air tracking from the gland down into the cervical tissues and mediastinum. Pneumomediastinum is potentially life-threatening and may lead to vessel compression and tamponade, or tracheal compression causing respiratory compromise [6]. Complications of pneumoparotitis include acute and chronic parotitis, abscess formation, fibrosis of the ducts, ductal incompetence, ductal dilation, and sialectasis [5].

Management strategies vary depending on stability of the patient and whether it is the first occurrence. For the first episode, non-surgical measures including hydration, massage, anti-inflammatory drugs, and warm compresses are often sufficient [6]. Prophylactic antibiotics are indicated to prevent superimposed infection [2]. Recurrence rates have been cited at 42.6% however avoidance of causative behaviors could prevent additional episodes [2]. For recurrent pneumoparotitis or complicated cases, surgical intervention may be warranted. Parotidectomy is the standard of care for recurrent infection. Parotid duct ligation has been suggested as an alternative, though risks abscess formation when performed in a contaminated field [6]. The patient described in this report did well with prophylactic antibiotics and has not returned to clinic with any signs of recurrence.

## Conclusions

History and physical exam findings consistent with pneumoparotitis with concomitant evidence of crepitus in the neck and chest should prompt suspicion and workup for pneumomediastinum. Though pneumoparotitis is rare, it is a well-established consequence of reflux of air and oral flora into Stensen’s duct as a result of elevated intraoral pressures, however, pneumomediastinum secondary to pneumoparotitis is not as well documented and could pose particular danger to patients if not recognized. While mild cases are likely appropriate for conservative management with prophylactic antibiotics in close consultation with otolaryngology and thoracic surgery colleagues, little information is available on the risks of pneumomediastinum secondary to pneumoparotitis or for how to best counsel patients regarding treatment options, particularly for severe cases. 
